# Bacteremia and Community-Acquired Pneumonia Caused by *Pantoea stewartii* Subspecies *indologenes*, Australia

**DOI:** 10.3201/eid3102.240546

**Published:** 2025-02

**Authors:** Lawrence Huang, Erin P. Price, Derek S. Sarovich, Dean Johns, Shradha Subedi

**Affiliations:** Sunshine Coast University Hospital, Birtinya, Queensland, Australia (L. Huang, D. Johns, S. Subedi); University of the Sunshine Coast Centre for Bioinnovation, Sippy Downs, Queensland, Australia (E.P. Price, D.S. Sarovich); Sunshine Cost Health Institute, Birtinya (E.P. Price, D.S. Saraovich, S. Subedi); The University of Queensland Centre for Clinical Research, Brisbane, Queensland, Australia (S. Subedi)

**Keywords:** pneumonia, bacteria, *Pantoea*, *Pantoea stewartii* subspecies *indologenes*, bacteremia, community-acquired pneumonia, Australia

## Abstract

We report infection with the phytopathogen *Pantoea stewartii* subspecies *indologenes* in a macadamia farmer from southeast Queensland, Australia. The patient had bloodstream infection and pneumonia develop after an unidentified inoculation event. Investigation determined that the most likely mode of transmission was inhalation from an environmental source on the farm.

*Pantoea* species are ubiquitous bacteria in both terrestrial and aquatic environments and have been isolated in animals, insects, and humans, although most of the 31 recognized species (*1*) are associated with plants (*2*). *Pantoea* species previously reported in human infections include *P. agglomerans*, *P. ananatis*, *P. brenneri*, *P. calida*, *P. conspicua*, *P. dispersa*, *P. eucrina*, *and P. septica*; *P. agglomerans* has been the most common (*2*).

*P. stewartii*, the cause of Stewart’s wilt in sweet corn and maize, was first discovered in the late 1890s. Researchers proposed 2 subspecies in 1993 based on host range: *stewartii* and *indologenes* (*3*). Unlike subspecies *stewartii*, subspecies *indologenes* is nonpathogenic to corn, instead causing disease in other agronomically significant crops, such as foxtail millet, pearl millet, and onions. Because of the risk this organism (particularly subspecies *stewartii*) poses to economically critical crops, many countries classify *P. stewartii* as a quarantine organism (*4*,*5*).

In 2022, researchers reported infection with *P. stewartii* in a human, initially identifying the species with moderate confidence as *P. septica* but then recategorizing the species designation based on 16S ribosomal RNA gene sequencing (*1*). We report phytopathogen *P. stewartii* subsp. *indologenes* infection in a macadamia farmer from southeast Queensland, Australia.

## The Study

**Figure 1:**
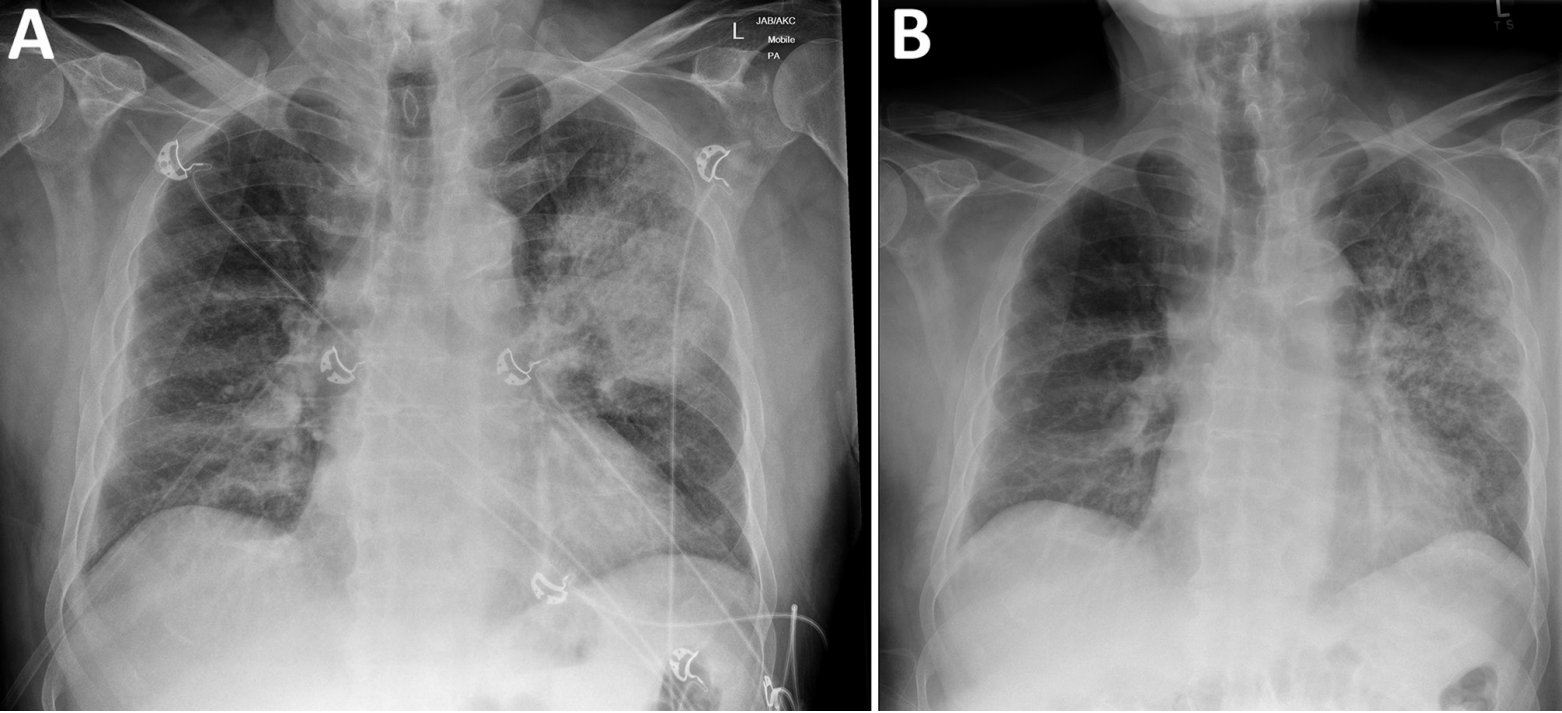
Chest radiographs of patient with *Pantoea stewartii* subspecies *indologenes* infection, Queensland, Australia. A) Radiograph at time of initial emergency department visit, showing dense left upper lobe consolidation consistent with pneumonia. B) Repeat radiograph on day 12 of hospital admission, showing resolving left upper and middle zone opacification.

In summer 2021, an 82-year-old man sought care at the emergency department of Sunshine Coast University Hospital (Birtinya, QLD, Australia), for sudden onset of fever, myalgia, arthralgia, nonproductive cough, and shortness of breath. The patient had recently moved to a macadamia farm in the Sunshine Coast hinterland and had previously resided on a pineapple farm in the same region for several decades. He reported no overseas travel history, no preceding history of inoculating injury from plant material, no recent skin wounds or infections, and no direct zoonotic contacts. Chest radiograph (Figure 1, panel A) identified moderately severe community-acquired multilobar pneumonia, with dense consolidation in the left upper lobe. The patient deteriorated rapidly because of septic shock, necessitating intubation and ventilation, and was transferred to the intensive care unit on day 1 of admission.

**Figure 2:**
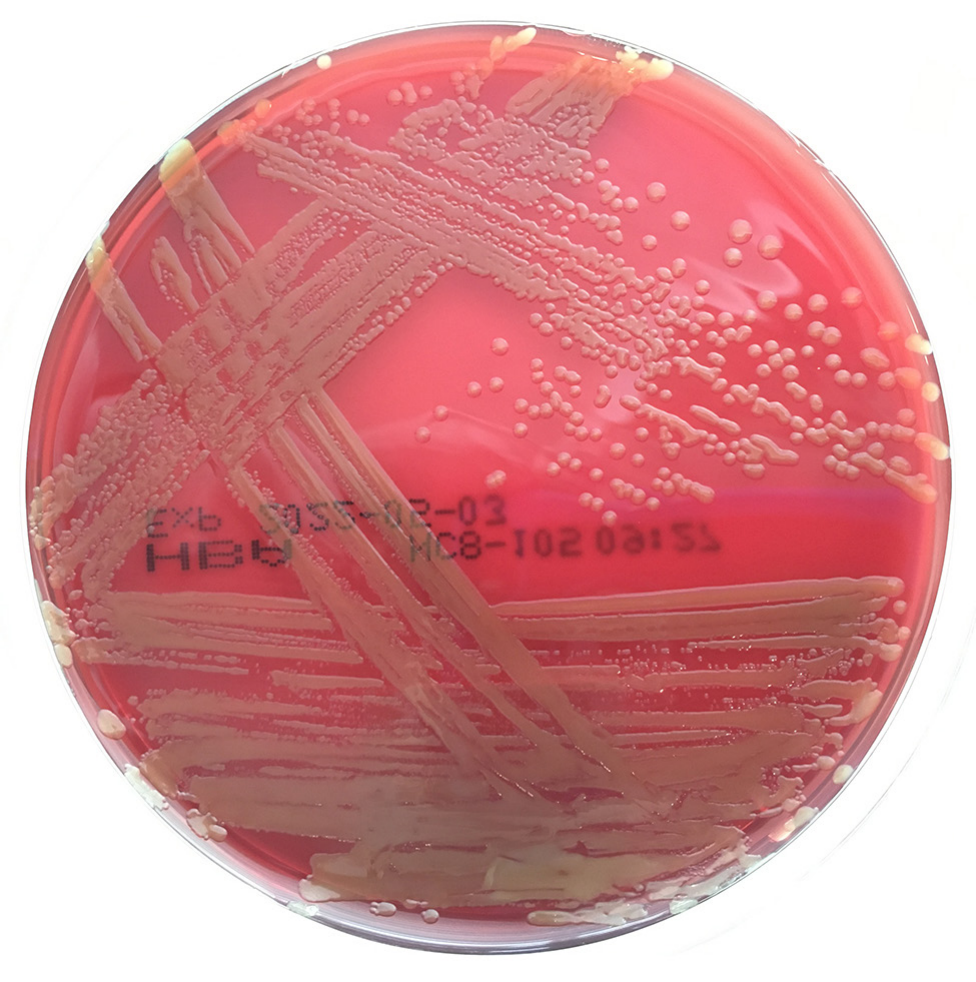
Results of blood culture for patient with *Pantoea stewartii* subspecies *indologenes* infection, Queensland, Australia. Yellow pigmented colonies grew on horse blood agar on day 1 of subculture after incubation in 5% CO_2_ at 35°C. VITEK MS matrix-assisted laser desorption/ionization time-of-flight mass spectrometry (bioMérieux, https://www.biomerieux.com) identified the pathogen as *Pantoea ananatis* with 99.9% probability, but comparative genome analysis revealed that the pathogen was most closely related to *P. stewartii* subsp. *indologenes*.

We observed lactose-fermenting, nonmucoid, yellow-pigmented colonies cultivated on MacConkey and horse blood agars (Figure 2) from blood culture (isolate SCHI0154.S.1), tracheal aspirate, and bronchial washing specimens. We determined the colonies to be catalase positive, oxidase negative, and spot indole positive. VITEK MS matrix-assisted laser desorption/ionization time-of-flight mass spectrometry (bioMérieux, https://www.biomerieux.com) revealed *P. ananatis* with 99.9% probability. The VITEK GN ID card identified the respiratory isolates as *Pantoea* sp. with 95% probability. SCHI0154.S.1 was resistant to ampicillin (MIC = 16 mg/L) and cephazolin (MIC ≥64 mg/L) but susceptible to amoxicillin/clavulanate (MIC ≤2 mg/L), ceftriaxone (MIC ≤1 mg/L), ciprofloxacin (MIC ≤0.25 mg/L), gentamicin (MIC ≤1 mg/L), meropenem (MIC ≤0.25 mg/L), piperacillin/tazobactam (MIC = 2 mg/L), and trimethoprim/sulfamethoxazole (MIC ≤20 mg/L).

We treated the patient with intravenous amoxicillin/clavulanate (2.2 g/8 h) for a total of 10 days and successfully extubated him on day 7. Chest radiograph opacification on day 12 showed improvements (Figure 1, panel B). At outpatient follow-up, the patient had reached full recovery.

To permit accurate speciation and genetic comparison with other *Pantoea* isolates, we compared Illumina NovaSeq 2 × 150bp whole-genome sequencing reads generated for SCHI0154.S.1 (Australian Centre for Ecogenomics, St Lucia, Queensland, Australia) against 26 *Pantoea* spp. reference genomes (Appendix Table). We then analyzed those sequencing results against 48 publicly available *P. stewartii* genomes (Appendix Table). We carried out phylogenomic reconstruction of orthologous, biallelic single-nucleotide polymorphisms by using default SPANDx v4.0.3 (*6*) settings and PAUP* v4.0a.168 (*7*). We performed bootstrapping using 1,000 re-samples on the *P. stewartii* tree to assess clade confidence. We visualized trees in FigTree v1.4.0. We deposited the SCHI0154.S.1 assembly into GenBank (accession no. GCA_030144305.1).

Phylogenomic analysis revealed that SCHI0154.S.1 was most closely related to *P. stewartii* subsp. *indologenes* PANS 07–14 (Appendix Figure), which was isolated from a Verbena plant on an onion farm in Georgia, USA, in 2007 (*8*). SCHI0154.S.1 and PANS 07–14 differed by 1,015 single-nucleotide polymorphisms. In contrast, the only other genome-sequenced *P. stewartii* isolate from Australia, C10109_Jinnung (also subsp. *indologenes*), retrieved from a sick, captive western ground parrot (*Pezoporus flaviventris*), at Perth Zoo (Perth, WA, Australia) in 2021 (*9*), differed from SCHI0154.S.1 by 27,876 single-nucleotide polymorphisms.

One previous study reported *P. stewartii* associated with a human infection, with taxonomic assignment based on genetic similarity analysis of an unpublished 1,212-bp 16S ribosomal RNA amplicon reported to be 99.69% similar to *P. stewartii* strain 08BF11TN (GenBank accession KX146472.1) (*1*). To confirm this result, we repeated an ‘All genomes’ National Center for Biotechnology Information BLAST v2.15.0+ (https://blast.ncbi.nlm.nih.gov) analysis of the 1,451bp KX146472.1 sequence on August 16, 2024, using both the ‘Complete, Microbes’ and ‘Draft, Microbes’ databases. We restricted search parameters to ‘*Pantoea* (taxid:53335)’ and ‘megablast’. Our BLAST analysis found a closer match to *P. agglomerans* 33.1 (accession no. NZ_CP083809.1; 99.45% identity and 100% query coverage) than to *P. stewartii* RON18713 (accession no. NZ_CP116285.1; 98.07% identity and 100% query coverage) using the ‘Complete, Microbes’ genome database. Similarly, the same BLAST search using the ‘Draft, Microbes’ genome database identified a closer match to *P. vagans* 848 (accession no. JUQR01000382.1; 99.79% identity and 100% query coverage) and *P. septica* FF5 (accession no. CCAQ010000001.1; 99.72% identity and 100% query coverage) than to *P. stewartii* (best hit was strain RSA36 [accession no. LDSK01000027.1]; 98.00% identity and 100% coverage).

## Conclusions

This unusual case confirms that the phytopathogen *P. stewartii* can cause life-threatening infections in humans. Although 1 published study described *P. stewartii* bacteraemia after a poststroke stent implantation in a patient from Spain (*1*), our repeat analysis suggested that this previously reported case was more likely caused by *P. septica* or *P. agglomerans*.

Because almost nothing is known about *P. stewartii* disease in humans or potential virulence factors of *P. stewartii* and its subspecies, this organism might be clinically underdiagnosed by current diagnostic methods, being misidentified as other more familiar *Pantoea* species. In support of this hypothesis, previously described *P. agglomerans* clinical isolates deposited into type culture collections have been reclassified as *P. ananatis*, *Erwinia* spp., or *Enterobacter* spp. on the basis of housekeeping gene sequencing (*10*). Further complicating matters, many taxonomic reassignments have occurred within the Erwiniaceae family in recent decades, making it challenging to track potential historical reports of *P. stewartii* human infection.

In our study, once the farmer’s infection was confirmed to be *P. stewartii*, we conducted a subsequent thorough clinical history to determine the likely source and mode of transmission. The patient reported no previous history of travel outside of Australia or recent injuries suggestive of dissemination from skin inoculation.

We noted just 1 previously report of *P. stewartii* in Australia, detected in a critically endangered native parrot that fell gravely ill in captivity (*9*), suggesting that birds may represent an underappreciated reservoir for *P. stewartii* subsp. *indologenes* global dissemination. However, in that case, the authors reported a link to parrot pellets commercially imported from the United States (*9*). It is therefore possible that *P. stewartii* was introduced into eastern Australia, and then to our patient’s macadamia farm, through a commercially imported agricultural product originating from the United States. In our study, although we could not determine the precise source of infection from the research conducted, the farmer’s clinical features (i.e., pneumonia with subsequent hematogenous dissemination), lack of clear inoculation source, and limited travel suggested the most likely mode of transmission to be inhalation from an environmental source on the farm.

Appendix Additional information for bacteremia and community-acquired pneumonia caused by *Pantoea stewartii* subspecies *indologenes*, Australia.
