# Supplementary material for: Bacteremia and Community-Acquired Pneumonia Caused by Pantoea stewartii Subspecies indologenes, Australia
Source: Emerg Infect Dis. 2025 Feb;31(2):328–31. doi: 10.3201/eid3102.240546 (PMC11845149; doi:10.3201/eid3102.240546)
Supplement: Appendix — Additional information for bacteremia and community-acquired pneumonia caused by Pantoea stewartii subspecies indologenes, Australia. [file 24-0546-Techapp-s1.pdf]

Article DOI: https://doi.org/10.3201/eid3102.240546

EID cannot ensure accessibility for supplementary materials supplied by authors. Readers who have difficulty accessing supplementary content should contact the authors for assistance.

Bacteremia and Community-Acquired Pneumonia Caused by *Pantoea stewartii* Subspecies *indologenes*, Australia

Appendix

Appendix Table. *Pantoea* genomes examined in a study of bacteremia and community-acquired pneumonia caused by *Pantoea stewartii* subspecies *indologenes*, Australia.

Strain	Collection date	Origin	GenBank accession
Pantoea stewartii			
P. stewartii subsp. unknown 626	Unknown	Maize, Madras, India	GCA_013277595.1
P. stewartii subsp. unknown CCM 8557	2021	Unknown	GCA_042681965.1
P. stewartii subsp. unknown M009	2013	Waterfall, Malaysia	GCA_000786255.1
P. stewartii subsp. unknown M073a	2013	Waterfall, Malaysia	GCA_000803205.1
P. stewartii subsp. unknown NC66	2019	Human nasal swab, Assam, India	GCA_035787335.1
P. stewartii subsp. unknown NRRL B-133	1924	Pineapple, Philippines	GCA_014218605.1
P. stewartii subsp. *indologenes* C10109_Jinnung	2021	Western ground parrot faeces, WA, Australia	GCA_029991035.1
P. stewartii subsp. *indologenes* ICMP 10132	2022	Sugar cane, Brazil	GCA_029433915.1
P. stewartii subsp. *indologenes* LMG 2630	2019	Guar, HI, USA	GCA_030336395.1
P. stewartii subsp. *indologenes* LMG 2632	1960	Foxtail millet, India	GCA_000757405.2
P. stewartii subsp. *indologenes* LMG 2671	2019	Pineapple, HI, USA	GCA_030370575.1
P. stewartii subsp. *indologenes* NCPPB 1562	1963	Pearl millet, India	GCA_017051845.1
P. stewartii subsp. *indologenes* NCPPB 1877	1966	Guar pulse, HI, USA	GCA_017051875.1
P. stewartii subsp. *indologenes* NCPPB 2275	1970	Pearl millet, India	GCA_017051895.1
P. stewartii subsp. *indologenes* NCPPB 2281	1970	Foxtail millet, India	GCA_017051805.1
P. stewartii subsp. *indologenes* NCPPB 2282	1956	Pearl millet, India	GCA_017051815.1
P. stewartii subsp. *indologenes* PANS 07–4	2007	Foxtail millet, GA, USA	GCA_017052095.1
P. stewartii subsp. *indologenes* PANS 07–6	2007	Corn, GA, USA	GCA_017052115.1
P. stewartii subsp. *indologenes* PANS 07–10	2007	Pearl millet, GA, USA	GCA_017051975.1
P. stewartii subsp. *indologenes* PANS 07–12	2007	Pearl millet, GA, USA	GCA_017052015.1
P. stewartii subsp. *indologenes* PANS 07–14	2007	Verbena, GA, USA	GCA_017051935.1
P. stewartii subsp. *indologenes* PANS 99–15	1999	Crab grass, GA, USA	GCA_017051945.1
P. stewartii subsp. *indologenes* PNA 03–3	2003	Onion, GA, USA	GCA_003201175.1
P. stewartii subsp. *indologenes* PNA 14–9	2014	Onion, GA, USA	GCA_017052375.1
P. stewartii subsp. *indologenes* PNA 14–11	2014	Onion, GA, USA	GCA_017052195.1
P. stewartii subsp. *indologenes* PNA 14–12	2014	Onion, GA, USA	GCA_017052135.1
P. stewartii subsp. *indologenes* PNA 15–2	2015	Onion, GA, USA	GCA_017052175.1
P. stewartii subsp. *indologenes* RON18713	2020	Brazil nut tree leaves, Rondônia, Brazil	GCA_030064655.1
P. stewartii subsp. *indologenes* SCHI0154.S.1	2022	Human blood, QLD, Australia	GCA_030144305.1
P. stewartii subsp. *indologenes* SJM_1_1	2022	Bougainvillea glabra leaves, Haikou, People's Republic of China	GCA_040746145.1
P. stewartii subsp. *indologenes* ST25	2020	Rice seeds, TX, USA	GCA_025599245.1
P. stewartii subsp. *indologenes* ZJ-FGZX1	2017	Lucky bamboo, Guangdong, People's Republic of China	GCA_011044475.1
P. stewartii subsp. *stewartii* 34258,2/15	2015	Sweet corn leaves, Emilia-Romagna, Italy	GCA_030336485.1
P. stewartii subsp. *stewartii* 34596,1/15	2015	Sweet corn leaves, Emilia-Romagna, Italy	GCA_030336515.1
P. stewartii subsp. *stewartii* 49472,2/18	2018	Sweet corn leaves, Emilia-Romagna, Italy	GCA_030370555.1

Strain	Collection date	Origin	GenBank accession
P. stewartii subsp. *stewartii* 49474,1/18	2018	Sweet corn leaves, Emilia-Romagna, Italy	GCA_030370545.1
P. stewartii subsp. *stewartii* A206	2011	Infected leaves, peach palm, Costa Rica	GCA_001310285.1
P. stewartii subsp. *stewartii* CCUG 26359	1941	Sweet corn, IA, USA	GCA_008801695.1
P. stewartii subsp. *stewartii* CREA-DC 1788	<2000	Corn, Roma, Italy	GCA_030370565.1
P. stewartii subsp. *stewartii* DC283	1967	Maize, IL, USA	GCA_002082215.1
P. stewartii subsp. *stewartii* HR3–48	Unknown	Rice, Sanya, People's Republic of China	GCA_025765915.1
P. stewartii subsp. *stewartii* IPV-BO 2766	1994	Sweet corn, Lombardia, Italy	GCA_030370585.1
P. stewartii subsp. *stewartii* ICMP 257	2002	Sugar cane, Brazil	GCA_042665675.1
P. stewartii subsp. *stewartii* MS1	2017	Jackfruit, Pahang, Malaysia	GCA_010273335.1
P. stewartii subsp. *stewartii* NS381	2013	Rice, India	GCA_001476355.1
P. stewartii subsp. *stewartii* RSA13	2013	Rice, India	GCA_001477215.1
P. stewartii subsp. *stewartii* RSA30	2013	Rice, India	GCA_001476795.1
P. stewartii subsp. *stewartii* RSA36	2013	Rice, India	GCA_001476375.1
P. stewartii subsp. *stewartii* S301	2011	Infected leaves, peach palm, Costa Rica	GCA_001310295.1
Other related species			
[*Curtobacterium*] *plantarum* LMG 16222	~1989	Soybean leaves, USA	GCA_003710245.1
[*Erwinia*] *mediterraneensis* Marseille-P5165	Unknown	Human skin, Senegal	GCA_900604315.1
Pantoea agglomerans FDAARGOS 1447	1956	Knee laceration, UK	GCA_019048385.1
Pantoea alhagi LTYR-11Z	2014	Leaf, Xinjiang province, People's Republic of China	GCA_002101395.1
Pantoea allii PNA 200–10	Unknown	Georgia, USA	GCA_003148935.1
Pantoea ananatis PA13	Unknown	Diseased rice grain, South Korea	GCA_000233595.1
Pantoea anthophila 11–2	2012	Hypersaline lake water, Hawai'i, USA	GCA_000969395.1
Pantoea brenneri IIFCSG-B1	2015	International space station, USA	GCA_013403315.1
Pantoea coffeiphila 1480	Unknown	Unknown, Nebraska, USA	GCA_016909495.1
Pantoea conspicua LMG 24534	2008	Human blood, Paris, France	GCA_002095315.1
Pantoea cypripedii WS4375	Unknown	Unknown	GCA_017875755.1
Pantoea deleyi LMG 24200	2007	Diseased eucalyptus leaves, Uganda	GCA_022647325.1
Pantoea dispersa Lsch	2019	Drinking water, Lao People's Democratic Republic	GCA_019890955.1
Pantoea endophytica 596	2013	Maize, Beijing, People's Republic of China	GCA_002858935.1
Pantoea eucalypti LMG 24197	Unknown	Diseased eucalyptus leaves, Uruguay	GCA_009646115.1
Pantoea eucrina XL123	2018	Cucumber rhizosphere, Nanjing, People's Republic of China	GCA_020079945.1
Pantoea latae AS1	2015	Cycad, Florida, USA	GCA_002077695.1
Pantoea phytobeneficialis MSR2	2015	Mimosa scabrella, Florianopolis, Brazil	GCA_009728735.1
Pantoea piersonii IIIF1SW-P2	2016	International space station, USA	GCA_003612015.1
Pantoea pleuroti JZB 2120015	2013	King trumpet mushroom, Beijing, People's Republic of China	GCA_014156615.1
Pantoea rodasii DSM 26611	2007	Diseased eucalyptus seedlings, Bogotá, Columbia	GCA_002811195.1
Pantoea rwandensis LMG 26275	2005	Diseased eucalyptus seedlings, Rwanda	GCA_002095475.1
Pantoea septica MGYG-HGUT-02423	Unknown	Human gut, USA	GCA_902386985.1
Pantoea vagans LMG 24199	Unknown	Diseased eucalyptus seedlings, Uganda	GCA_004792415.1
Pantoea wallisii LMG 26277	2006	Diseased eucalyptus seedlings, South Africa	GCA_002095485.1

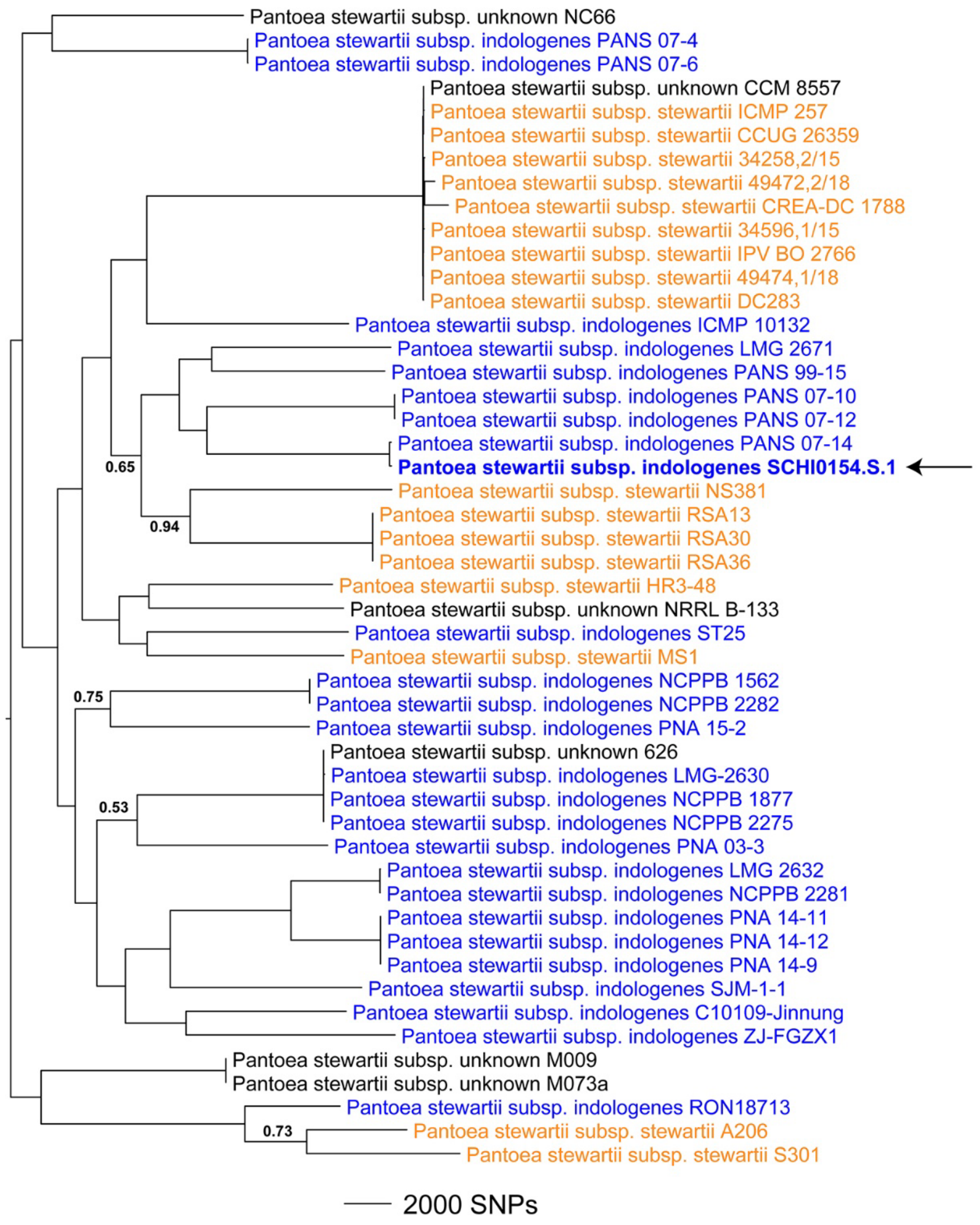

Appendix Figure. Midpoint-rooted maximum parsimony phylogenomic analysis of an Australian clinical strain, SCHI0154.S.1 (bolded), against 48 other *Pantoea stewartii* strains (Appendix Table), to determine the closest strain relative/s and potential origin of infection. This tree was reconstructed using 98,746 orthologous, biallelic, genome-wide single-nucleotide polymorphisms identified by SPANDx (*6*). The patient's isolate was most closely related to PANS 07–14, isolated from a verbena plant in Georgia, USA, in 2007 (*8*). Notably, the only other Australian strain, C10109_Jinnung (*5*), was genetically unrelated to SCHI0154.S.1. Branches with <100% bootstrap support are labeled, except within the *P. stewartii* subsp. *stewartii* clade, due to limited resolution among these strains. Consistency index = 0.39.
